# Supplementary material for: Modeling glioblastoma heterogeneity as a dynamic network of cell states
Source: Mol Syst Biol. 2021 Sep 16;17(9):e10105. doi: 10.15252/msb.202010105 (PMC8444284; doi:10.15252/msb.202010105)
Supplement: Supplementary file 6 — Source Data for Figure 5 [file MSB-17-e10105-s004.zip › Figure5A_sourcedata/GSEA_3017/hallmarks_stateC.GseaPreranked.1621934520804/HALLMARK_UV_RESPONSE_DN.html]

Details for gene set HALLMARK\_UV\_RESPONSE\_DN[GSEA]

|  || Dataset | state13017 |
| Phenotype | NoPhenotypeAvailable |
| Upregulated in class | na\_neg |
| GeneSet | HALLMARK\_UV\_RESPONSE\_DN |
| Enrichment Score (ES) | -0.4952111 |
| Normalized Enrichment Score (NES) | -1.9819044 |
| Nominal p-value | 0.0 |
| FDR q-value | 0.008104164 |
| FWER p-Value | 0.04 |
Table: GSEA Results Summary

  

Fig 1: Enrichment plot: HALLMARK\_UV\_RESPONSE\_DN      
 Profile of the Running ES Score & Positions of GeneSet Members on the Rank Ordered List

  

| PROBE | GENE SYMBOL | GENE\_TITLE | RANK IN GENE LIST | RANK METRIC SCORE | RUNNING ES | CORE ENRICHMENT || 1 | RND3 |  |  | 2 | 0.713 | 0.0890 | No |
| 2 | SERPINE1 |  |  | 323 | -0.286 | -0.4257 | No |
| 3 | DLC1 |  |  | 347 | -0.296 | -0.4269 | No |
| 4 | IGFBP5 |  |  | 384 | -0.311 | -0.4487 | No |
| 5 | SIPA1L1 |  |  | 412 | -0.329 | -0.4525 | Yes |
| 6 | LDLR |  |  | 419 | -0.334 | -0.4196 | Yes |
| 7 | MYC |  |  | 421 | -0.336 | -0.3778 | Yes |
| 8 | INSIG1 |  |  | 434 | -0.342 | -0.3541 | Yes |
| 9 | MAP1B |  |  | 443 | -0.348 | -0.3229 | Yes |
| 10 | ATXN1 |  |  | 486 | -0.392 | -0.3445 | Yes |
| 11 | F3 |  |  | 505 | -0.432 | -0.3195 | Yes |
| 12 | BDNF |  |  | 524 | -0.459 | -0.2911 | Yes |
| 13 | PLPP3 |  |  | 528 | -0.466 | -0.2359 | Yes |
| 14 | GJA1 |  |  | 552 | -0.527 | -0.2072 | Yes |
| 15 | ANXA2 |  |  | 563 | -0.567 | -0.1509 | Yes |
| 16 | HAS2 |  |  | 587 | -0.740 | -0.0946 | Yes |
| 17 | PMP22 |  |  | 590 | -0.837 | 0.0103 | Yes |
Table: GSEA details [plain text format]

  

Fig 2: HALLMARK\_UV\_RESPONSE\_DN: Random ES distribution      
 Gene set null distribution of ES for **HALLMARK\_UV\_RESPONSE\_DN**
